# Age-Related Patterns in the Subjective Appraisal of Cognition

**DOI:** 10.1093/geroni/igaa057.1163

**Published:** 2020-12-16

**Authors:** Claudia Jacova, Samantha Smith, Frank Robertson

**Affiliations:** 1 Pacific University, Hillsboro, Oregon, United States; 2 Student, Hillsboro, Oregon, United States

## Abstract

Subjective cognitive decline (SCD) is a construct of high interest in aging and dementia because individuals endorsing it are at higher risk of developing cognitive problems. It is unclear how individuals arrive at the judgement that they have SCD. Here we aimed to understand which SCD symptoms give rise to the perception of decline as older adults age. Community-dwelling adults (N=494, mean age=63.6, SD=5.44), completed the Subjective Cognitive Decline Questionnaire (SCD-Q) online, using an online crowdsourcing site. The SCD-Q consists of one global question regarding self-perceived decline (yes/no) and 24 questions about everyday functioning which we utilized to form a memory, language, and executive functioning domain score, higher for greater perceived decline. Logistic regression revealed that memory and language domains predicted the likelihood of endorsing SCD for adults aged >64 (Memory: OR=1.76, CI=1.47-2.05; Language: OR=1.66, CI=1.30-2.02). Only the memory domain predicted the likelihood of endorsing SCD for adults <63 (OR=2.69, CI=2.35-3.02). Executive functioning domain scores did not play a role in the relationship between SCD likelihood in either age group. The higher the self-perceived memory or language decline, the more likely older adults are to conclude they have SCD. Our results suggest there is an age-related trajectory in how people evaluate their cognition, with younger people only considering memory and older people considering both memory and language. Clinicians should be aware of this trajectory when examining patients with SCD. Executive functions should be specifically queried because they may not emerge from older adults’ self-reported cognitive problems.

